# Integrating an antiracism curriculum into public health core courses

**DOI:** 10.3389/fpubh.2026.1749330

**Published:** 2026-03-19

**Authors:** Teri E. Lassiter, Laura E. Liang, Rafael E. Pérez-Figueroa, Marian R. Passannante

**Affiliations:** School of Public Health, Rutgers University, Piscataway, NJ, United States

**Keywords:** academic public health, antiracism, curriculum, graduate training, racism

## Abstract

In public health, we are dedicated to training the next generation of leaders at local, national, and global levels. To achieve this goal, it was essential to integrate antiracist principles into our curricula and address issues related to racial justice, bias, white privilege, and systemic oppression. A report from the Association of Schools and Programs in Public Health (ASPPH) called on schools and programs of public health to “adopt and adapt public health curricula to highlight how racism and other forms of discrimination impact the health and well-being of populations and individuals.” Starting in the spring of 2024, our six core courses explored antiracism from various perspectives, fostering a comprehensive understanding of the topic. This interdisciplinary approach encouraged diverse viewpoints and facilitated dynamic discussions, emphasizing the interconnectedness of racism with all aspects of our society.

## 1 Introduction

Training the next generation of public health leaders requires more than technical knowledge—it involves preparing graduates to confront racism as a structural determinant of health. To do so, we must integrate the principles of antiracism into our curricula and explore issues related to racial justice, bias, white privilege, and systemic oppression (1, 2). Public health students and recent graduates have similarly called for re-envisioning curricula to embed antiracist and anti-oppressive frameworks across public health training (3).

Antiracism refers to the process of identifying, challenging, and changing the values, social structures, policies, and behaviors that perpetuate racism and racial inequities (4). There is a need to create a learning environment where students from all backgrounds have their voices heard, support authenticity and intersectionality, and foster consciousness among our students. This work can promote a culture of belonging, support authenticity and intersectionality, and promote growth by building a diverse pipeline of talent that can meet the future needs of public health. Integrating antiracism into the classroom provides opportunities “that foster equity, diversity, and inclusivity, and whether the assignments take into consideration the student story and experiences and promote critical thinking” (4). In response to this need, we developed and implemented an antiracism cross-cutting curriculum embedded across six core courses with the goal of equipping graduate students with the competencies to address racism and its links to public health, as well as creating an open space for discussions on the effects of institutional and structural racism.

Public health leaders encounter numerous challenges that impact our communities, often disproportionately affecting marginalized and minoritized populations. In 2018, the Wisconsin Public Health Association passed a resolution declaring racism a public health crisis, following a 2016 campaign against racism launched by Dr. Camara Jones, a former president of the American Public Health Association (APHA), in 2016. In response, state and local leaders also recognized racism as a public health crisis, emphasizing that “advancing racial equality and justice must be accompanied by the allocation of resources and strategic action” (5). In addition, the Association of Schools and Programs in Public Health (ASPPH) issued the report *Dismantling Racism and Structural Racism in Academic Public Health A Framework* in 2021 calling for schools of public health to (a)dopt and adapt public health curricula to highlight the ways racism and other forms of discrimination impact the health and well-being of populations and individuals (6).

Racism is recognized as a social determinant of health and a major contributor to health inequities in marginalized and minoritized communities (7, 8). Racism provides privilege to some groups while disadvantaging others. Jones (9) notes that racism “unfairly disadvantages some individuals and communities, unfairly advantages other individuals and communities, and saps the strength of the whole society through the waste of human resources.” Jones (10) identifies three levels of racism that impact society: institutional or structural racism, personally mediated racism, and internalized racism. Each illustrates how racism functions within our society and impacts the health of our nation. While *institutional* and *structural racism* emphasize somewhat different dimensions of systemic inequity (11), we use the term *structural racism* to encompass both for brevity.

Institutional or structural racism fundamentally causes racial health inequities; though rooted in history, it evolves over time to perpetuate conditions that lead to poor health for racially minoritized populations (12, 13). Structural racism manifests in various sectors, including education, housing, employment, healthcare, and the criminal justice system. According to Needham et al. (14), these policies and practices are embedded in our social institutions and undermine the growth and health of racial and ethnic individuals and communities by perpetuating policies that operate “as a system across multiple interconnected institutions”. Structural racism significantly affects the distribution of resources and access to opportunities. The consequences of structural racism reinforce unequal social, environmental, and economic conditions, which ultimately lead to poor health outcomes in minoritized populations. These populations face a disproportionate burden of environmental hazards (15–17), reduced access to healthcare, food, and transportation (16, 18–21), educational and employment opportunities (22, 23), recreation, and preventive health services (16, 17). Decades of racism and disinvestment in communities of color have led to residential segregation and housing inequities (16, 20–22), economic hardships (22, 24, 25), and the occurrence of other mechanisms such as political exclusion, incarceration, and state-sanctioned violence. The intent of this antiracism discourse is not to ascribe personal blame but to cultivate a shared sense of responsibility and agency to challenge and transform the systems that perpetuate inequities.

## 2 Understanding racism in public health

Racism has influenced public health policies and practices for over 400 years, with its roots tracing back to the mistreatment of enslaved Black individuals on southern plantations (17). The history of medicine and healthcare in the United States is closely linked to racism and the mistreatment of Black Americans and other minorities. These issues continue to impact modern medicine, as we can see in minority populations living in healthcare-shortage areas, where primary care physicians are scarce. In emergency rooms, these individuals often face neglect and do not receive the same treatment options available to the majority population.

In the 19th century, J. Marion Sims, often regarded as the father of modern gynecology, infamously conducted experiments on enslaved Black women to refine a surgical procedure for repairing vesicovaginal fistulas. These unethical practices continued with the infamous Tuskegee Syphilis Experiment, carried out by the U. S. Public Health Service, which studied the progression of untreated syphilis in African American men in rural Alabama. Racist policies have long affected public health and the public health system (17, 25). The exposure of the Tuskegee Study in 1972 ultimately led to the creation of The Belmont Report in 1979, which established ethical principles for conducting research involving human subjects (25). The publication of the book *The Immortal Life of Henrietta Lacks* (26) revealed that medical researchers collected cells from her body without her consent. These cells were used in medical research to develop treatments for sexually transmitted diseases, leukemia, influenza, and HIV/AIDS. Her family was not compensated until after her death (17). Modern health inequities were seen in 2022 when the COVID-19 pandemic swept across the country, and communities of color experienced higher rates of the virus and deaths due to already existing underlying health disparities and systemic barriers to care. Mistrust in public health institutions and policies once again came to the forefront as vaccines were not accessible in marginalized communities, resulting in lower rates of vaccine uptake.

## 3 Rationale for antiracism curriculum

Addressing racism in public health education is critical to advancing health equity and improving population health outcomes. Racism is a critical determinant of health inequities (27), influencing access to healthcare services (13), associated with multiple social determinants of health (28), and central to the lived experiences of communities facing health inequities (29). Despite its well-documented impact, most public health curricula often fail to comprehensively address racism as a structural determinant of health (30). Integrating antiracism education into public health training can equip future professionals with the knowledge and skills to challenge systemic inequities and advocate for policies that drive sustainable health outcomes.

Explicitly examining racism in public health training offers numerous advantages for students, faculty, and the broader community. For students, it can enhance critical thinking skills (31), foster cultural humility (32), and the ability to apply an equity-centered lens to public health practice (33). Faculty members can implement frameworks to address complex discussions about race and health inequities (34), reflect on existing biases (35), and enhance their teaching and research. Communities, particularly those disproportionately affected by health inequities (36), benefit from a workforce that is better prepared to implement inclusive, culturally appropriate, and responsive public health interventions. By embedding antiracism principles into curricula (37), public health schools and programs can train professionals equipped with the skills and knowledge to more effectively address health equity.

An antiracism curriculum aligns with the core mission and values of public health education (38), which emphasizes social justice, health equity, and community well-being. Public health schools and programs have a responsibility to train professionals who can address the root causes of health inequities (39), including structural and systemic racism. By institutionalizing antiracism education, public health schools and programs can demonstrate a commitment to advancing a healthy nation and society. This alignment not only strengthens academic programs but also reinforces the meaning of public health (20, 40): improving health outcomes for all populations, particularly those experiencing marginalization.

## 4 Initial implementation

**Figure 1:**
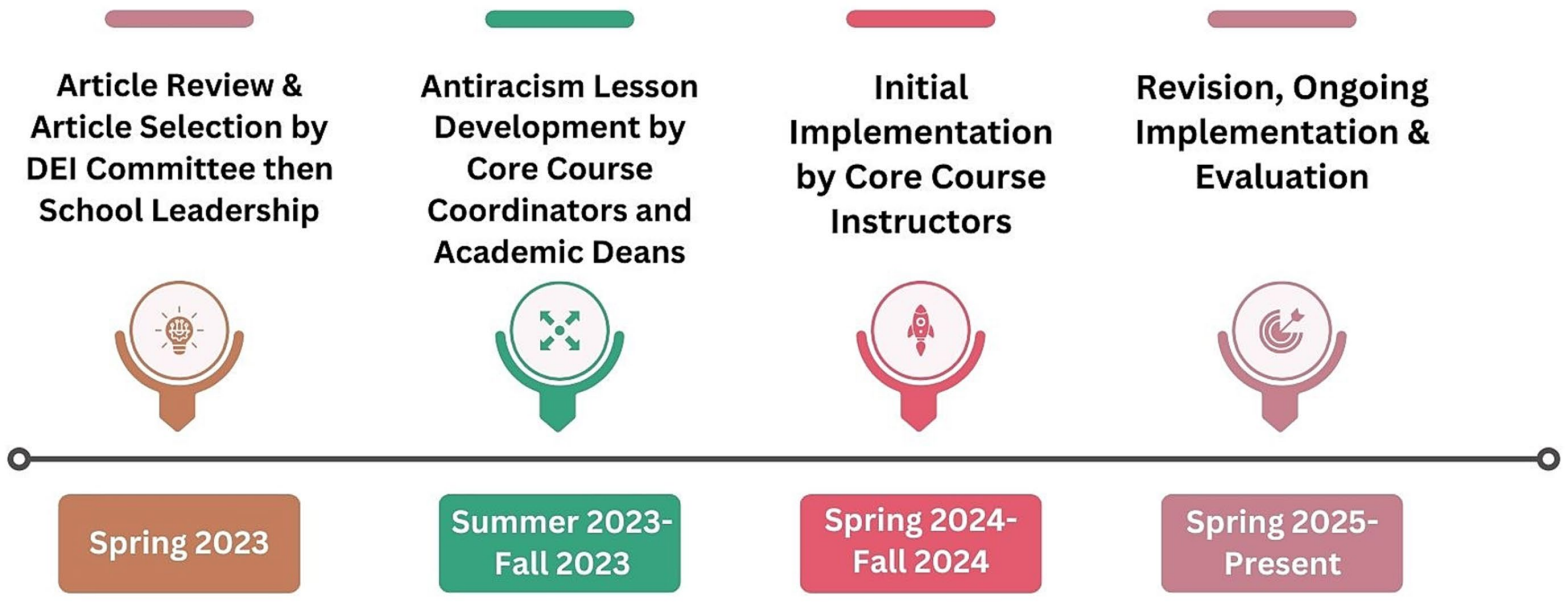
Timeline for antiracism curriculum implementation.

The implementation of the antiracism cross-cutting curriculum began with a rigorous review process. The implementation of the antiracism cross-cutting curriculum unfolded through a staged, school-wide review and planning process, summarized in the project timeline (Figure 1). The school’s Diversity, Equity, and Inclusion Committee, comprised of faculty, staff, and student representatives, identified and examined numerous scholarly articles that supported our efforts to increase student development around antiracism. Articles were selected based on their relevance to health outcomes shaped by racism, diversity of authorship, and inclusion of cross-cutting perspectives. The committee prioritized a balanced set of empirical and conceptual pieces representing data-driven analyses, environmental contexts, policy frameworks, and behavioral approaches, and excluded articles focused narrowly on a single topic or perspective. After extensive deliberation, the committee narrowed down the field to preferred and secondary articles, each offering unique perspectives and practical applications. These articles were presented to school leadership, who then selected a single, seminal article as the foundation for the initiative (see Table 1 for the list of preferred and secondary articles, as well as the final article selected for the curriculum). School leadership selected one article that could be integrated across all six core courses. Although the article was on the secondary article list, it aligned with an area of research excellence within our program and offered the strongest relevance to course content. This article (hereafter referred to as the “Stokes article”) became the central text around which core course coordinators would develop a tailored lesson focused on antiracism specific to their core course, ensuring alignment with each core course’s learning objectives within the MPH program. The Stokes article provided actionable entry points in each core course for classroom discussion and critical reflection, allowing students to examine institutional and structural racism through discipline-specific applications.

**Table 1:** Articles reviewed and recommended by the diversity, equity, and inclusion committee to school leadership.

Preferred articles
Braveman et al. (41)
Camara (10)
Krieger (42)
Wolitski et al. (43)
Yerger et al. (44)

Secondary articles
Halkitis et al. (45)
Hanna-Attisha et al. (46)
Johnson (47)
Stokes et al. (48)
Williams et al. (13)
Yearby (28)

Following the article selection, core course coordinators embarked on designing specific lesson plans and integrating the chosen article’s concepts into their respective curricula. Lesson plans were developed for the following courses: PHCO 0501 Health Systems and Policy, PHCO 0502 Principles and Methods of Epidemiology, PHCO 0503 Introduction to Environmental Health, PHCO 0504 Introduction to Biostatistics, PHCO 0505 Social and Behavioral Sciences in Public Health, and PHCO 0513 Leadership and Management Essentials for Public Health (see Table 2 for brief descriptions of the lesson for each core course). These graduate-level core courses are required for the MPH degree and are offered every term, typically with two or more sections per course; they are primarily lecture-based with embedded discussion and small group activity components, enroll approximately 15–54 students per section, and are coordinated by six core faculty members. The lesson plans varied based on the competencies and objectives of each core course, but all used the Stokes article as the context for learning. For example, the lesson plan for the biostatistics core course had students evaluate the data provided in the Stokes article and then asked them to consider the public health implications of the findings, while the lesson plan for the social and behavioral sciences core course had students focus on the implications of the Stokes article findings on the design of a health education campaign to prevent youth from using tobacco-related products.

**Table 2:** Antiracism lesson overview by core course.

Public health core course	Antiracism lesson focus
PHCO 0501—Health Systems and Policy	Students discussed racial and ethnic differences in tobacco use among youth to develop tobacco-control policy recommendations, considering urban, suburban, and rural contexts, identifying relevant government agencies, and outlining advocacy strategies with community partners.
PHCO 0502—Principles and Methods of Epidemiology	Students identified the epidemiological study design, described the key exposure-outcome association, and summarized main findings as a foundation for a discussion of racism and its implications for public health.
PHCO 0503—Introduction to Environmental Health	Students explored racism as an environmental and occupational health issue, emphasizing environmental justice, ethical responsibility, and the disproportionate impact on marginalized populations.
PHCO 0504–Introduction to Biostatistics	Students calculated proportions and confidence intervals across groups, then interpreted these results and reflected on how observed disparities can be understood within a social determinants framework.
PHCO 0505—Social and Behavioral Health Sciences in Public health	Students drew on study findings about racial/ethnic patterns in youth tobacco use to critique and redesign a youth-focused tobacco prevention print ad, generating recommendations grounded in health literacy, cultural awareness, and cultural competence.
PHCO 0513—Leadership and Management Essentials for Public Health	Students analyzed the lead author’s leadership approach, team-building decisions, and collaborative practices by reviewing study authorship, funding acknowledgments, and an interview with the lead author, then reflected on leadership style, inclusion, and implications for their own practice as public health leaders.

The core course coordinators, along with the associate deans for academic affairs and educational program development, met regularly as a team to review and provide feedback on the lessons being developed. To ensure consistency and effective implementation, instructors of the core courses participated in a training session before the semester they taught. This training session focused on the selected article, the developed lesson plan for their core course, and provided supplementary materials to enhance understanding and application. Additionally, a dedicated student module was created in the school’s Learning Management System, serving as an introductory platform. This module provided an integrated learning experience by offering an overview of antiracism from a public health perspective, outlining how antiracism would be integrated across the core courses, and including messaging from school leadership. Together, these materials established a shared conceptual foundation and positioned the Stokes article as a central reference point for course-specific application.

The mid-semester and end-of-semester course evaluations included additional questions specific to the antiracism cross-cutting curriculum. These questions aimed to gather feedback on students’ perceptions of the initiative, including its clarity, relevance, and impact on their learning. The evaluations offered valuable insights into students’ experiences, highlighting both strengths and areas for potential improvement. Collecting student feedback during and after implementing the antiracism curriculum ensured that the initiative could be refined and improved.

After the second semester of implementing the antiracism curriculum, further analysis was conducted through focus group sessions. Separate focus groups were held with core course instructors and students to explore their experiences in greater depth. These sessions provided a platform for open dialogue, allowing participants to share detailed insights, observations, and suggestions. The instructors’ focus groups centered on the effectiveness of the training, the implementation of lessons, and resource availability. The students’ focus groups examined their understanding of the central article, the integration of the lesson across courses, and the initiative’s overall impact on their learning and professional growth. These focus groups provided qualitative data that complemented the course evaluation findings, giving a comprehensive view of the initiative’s effectiveness and guiding future improvements.

Overall, initial feedback from both students and instructors was positive, with respondents highlighting the value of integrating antiracism content across the MPH core curriculum. Although we did not experience any significant resistance to the curricular changes, students noted challenges related to repeated engagement with the same central article across multiple courses. These formative findings informed iterative refinements to the initiative, including the use of multiple articles and the incorporation of videos to enhance engagement and reduce content fatigue. These evaluation activities provided actionable insights that continue to guide ongoing curricular improvement. Detailed quantitative and qualitative results are being prepared for dissemination in a separate manuscript.

## 5 Conclusion

It is essential to prepare today’s public health students to recognize and understand racism as a structural determinant of health, enabling them to effectively work with diverse racial and ethnic communities in today’s world. Integrating an antiracism section into our current curriculum provided students with the opportunity to explore this theme through a central article from the perspective of the six core courses. This approach introduced foundational concepts related to structural racism and health inequities within disciplinary contexts.

Moving forward, the antiracism curriculum will expand to include multiple articles and assignments tailored to each core course. This second phase will also incorporate videos, TedTalks^©^, and other interactive materials to enrich the learning experience. As the curriculum continues to evolve, it will be integrated into concentration courses to allow students to apply their knowledge to specific topic areas such as urban health, maternal child health, global health, and environmental health. We also acknowledge that, given the current political climate, not all public health programs or institutions are able to implement explicit antiracist curricular initiatives, underscoring the importance of institutional support in advancing this work.

## Data Availability

The original contributions presented in the study are included in the article/supplementary material, further inquiries can be directed to the corresponding author.
